# Surviving critical COVID-19: How functionality, physical, mental and cognitive outcomes evolve?

**DOI:** 10.1371/journal.pone.0284597

**Published:** 2023-06-23

**Authors:** Ana Teixeira-Vaz, José Afonso Rocha, Mafalda Oliveira, Tiago Simões-Moreira, David Almeida e Reis, Ana Isabel Silva, José Artur Paiva

**Affiliations:** 1 Physical Medicine and Rehabilitation Department, Centro Hospitalar Universitário de São João, Porto, Portugal; 2 Intensive Care Medicine Department, Centro Hospitalar Universitário de São João, Porto, Portugal; 3 Faculty of Medicine, University of Porto, Porto, Portugal; Federal University of Acre (UFAC), BRAZIL

## Abstract

**Purpose:**

To analyze the long-term consequences of critical COVID-19, regarding physical, mental, cognitive and functional impairments, and to describe its evolution through time.

**Methods:**

Prospective cohort study, with consecutive inclusion of patients admitted due to SARS-CoV-2 to intensive care units(ICU) of a tertiary-care center, between May/2020 and September/2021. All included patients were included in Physical and Rehabilitation Medicine(PRM) inpatient programs during ICU stay. Eligible patients were evaluated on PRM appointments 6 and 12 months after ICU discharge. In each visit, physical examination and a predefined set of scales were applied, aiming to comprehensively evaluate the three domains (physical, mental and cognitive) of post-intensive care syndrome and the patients’ functionality. Statistical analysis encompassed descriptive and univariate analysis.

**Results:**

A total of 42 patients were included: 66.7% males, mean age of 62 yo. In the physical domain, 6 months after ICU discharge, there was a significant reduction in quality of life (p-value = 0.034), muscle strength (p-value = 0.002), gait ability (p-value<0.001) and balance (p-values<0.001) and increased fatigue levels (p-value = 0.009), in comparison with reference values. Yet, a significative positive evolution was observed in all referred subdomains (p-values<0.05). Nevertheless, 12 months after discharge, muscle strength (p-value = 0.001), gait (p-value<0.001) and balance (p-value<0.001) were still significantly compromised. Regarding the mental domain, both at 6 and 12 months after discharge, the levels of anxiety and depression were significantly increased (p-values<0.001). Nonetheless, a positive evolution was also found (p-values<0.02). Cognitive performance was significantly impaired in comparison with reference values, both at 6 and 12 months (p-value<0.001). Yet, a global improvement was also depicted (p-value = 0.003). Six months after ICU discharge, 54.8% were autonomous in activities of daily living, a value that improved to 74.0% in the subsequent 6 months (p-value = 0.002).

**Conclusion:**

Critical COVID-19 survivors present significant physical, mental and cognitive impairments 6 and 12 months after ICU discharge, despite their positive evolution through time.

## Introduction

Coronavirus Disease 2019 (COVID-19), a primarily respiratory disease caused by the SARS-CoV-2 virus, firstly emerged in December 2019, with a report of severe flu-like illness in China [1]. After the disease spread to over 110 countries, a global pandemic was declared in March 2020 and as of that date the number of cases has been increasing daily, posing a severe health threat at a global scale [2]. Worldwide, there have been more than 759 408 703 confirmed cases, including 6 866 434 deaths [2]. Specifically in Portugal, there have been 5 570 473 confirmed cases of COVID-19 with 26 266 deaths [2].

Most infected patients are asymptomatic or paucisymptomatic. Nevertheless, up to 15% have severe disease requiring admission to intensive care units (ICU). In these cases, appropriate critical care delivery is a cornerstone to reduce mortality, which reaches 75% in some series [3].

Rightfully, the initial focus of COVID-19 research was on acute treatment. However, after three years, the high number of critical COVID-19 survivors has raised emerging questions about mid and long-term outcomes [4, 5].

Regardless of the primary disease, survivors of a prolonged stay in the ICU may experience mid and long-term complications related to the critical illness, to the therapy and to the ICU environment itself [6]. Post-Intensive Care Syndrome (PICS) is defined as new or worsening physical, mental and cognitive disorders that negatively affect daily functioning and quality of life (QoL) in survivors of critical illness [7, 8]. This syndrome seems to be prevalent and impactful in critical COVID-19 survivors, at least in the first year after ICU discharge [6, 9].

A comprehensive description of these patients’ follow-up is essential to further assist the design and implementation of rehabilitation interventions and long-term care management for individuals with PICS secondary to COVID-19. Indeed, some previous investigations have addressed the mid and long-term disabilities of critical COVID-19 survivors [10–13]. Yet, there is still a substantial gap in knowledge regarding the extent of the impairments and their evolution through time, specifically in the physical domain and functionality. Also, the characteristics of Physical and Rehabilitation Medicine (PRM) interventions and their impact on the patient’s trajectory also require further clarification.

As so, the primary aim of this study was to describe the long-term consequences of critical COVID-19, regarding physical, mental, cognitive and functional impairments, as well as its evolution through time. As a secondary goal, we intended to characterize PRM intervention in this subset of patients.

## Materials and methods

### Ethics committee approval statement

This study has been approved by our institutional research ethics committee before started (*Comissão de Ética para a Saúde do Centro Hospitalar Universitário de São João; number of approval*: *22/21)*, and it has been conducted in accordance with the principles set forth in the Helsinki Declaration. Written informed consent was obtained from all patients.

### Study design

Prospective cohort study with consecutive inclusion of patients admitted to one of four ICU of an Intensive Care Department in a tertiary-care center, between May/2020 and September/2021.

Inclusion criteria were age ≥ 18 years old and ICU admission diagnosis of acute respiratory distress syndrome (ARDS) due to SARS-CoV-2, requiring invasive mechanical ventilation (IMV) for ≥ 48 hours (h). Patients who died during ICU stay were excluded.

After hospital discharge, all patients were reached by telephone and asked to attend a specific post-COVID-19 PRM outpatient appointment. When it was not possible to contact the patients through a single telephonic contact, five more attempts, in different days and at different day hours (from 10am to 5pm, on weekdays) were performed. First PRM appointment was scheduled at six months after ICU discharge. A re-evaluation visit was performed at 12 months after ICU discharge. If any of the appointments were missed, the patient was given two opportunities to reschedule in a one-month period.

The appointments were performed at the outpatient clinic of the PRM department of our center. All participants were interviewed face-to-face by the same PRM physician.

### Definitions

ARDS was defined in accordance with the Berlin definition, as an acute syndrome of lung inflammation and increased alveolar-capillary permeability associated with severe hypoxia and bilateral infiltrates on chest radiographs, without evidence of left heart failure [14].

A COVID-19 ARDS case was assumed when a positive result on real-time reverse-transcriptase–polymerase-chain-reaction (RT-PCR) assay of nasal and pharyngeal swab specimens was depicted in the first 24h after hospital admission.

### Data collection and outcome measures

Data regarding socio-demographic characteristics, functional status, comorbidities, characteristics of the critical respiratory illness and complications during ICU stay were collected by the main investigator from the electronic clinical records (ECR) using a predefined form.

Comorbidities included hypertension, diabetes mellitus, hyperlipidemia, obesity, smoking habits, atrial fibrillation, ischemic heart disease, heart failure, peripheral vascular disease, chronic pulmonary obstructive disease, asthma, sleep apnea, psychiatric pathology, oncologic pathology and immunosuppression.

To describe the characteristics of the critical respiratory illness, information regarding severity scores at ICU admission, namely Acute Physiology and Chronic Health Evaluation (APACHE) and Simplified Acute Physiology Score II (SAPS II) was retrieved. Additionally, data regarding the number of days at the ICU and total length of stay, number of days under IMV, need and number of days under vasopressors, renal replacement therapy, extracorporeal membrane oxygenation (ECMO) and the need for prone positioning were also collected. Several potential complications during ICU stay were assessed, namely neurological, abdominal, cardiovascular, cutaneous and infectious. Neurological complications (signs, symptoms and syndromes) comprised aphasia, dysarthria, dysphonia, dysphagia, focal weakness, delirium, seizures, cerebrovascular diseases, encephalopathy, encephalitis, myelitis, peripheral neuropathies and signs of corticospinal tract dysfunction (CSTD). Cardiovascular complications included bradyarrhythmia, tachyarrhythmia (atrial fibrillation, flutter, others), tachycardia-bradycardia syndrome, secondary myocardial injury, cardiac arrest, pericarditis, pericardial effusion, endocarditis, acute heart failure and cardiogenic chock. Abdominal complications encompassed hepatitis, gastrointestinal bleeding, pseudo-obstruction and obstruction, diarrhea and constipation. Skin complications included the presence of pressure injuries. Infectious complications were considered in the presence of ICU-acquired infections, superimposed to the primary infectious diagnosis.

Data regarding the aforementioned characteristics was gathered on a database where each patient received a code number to secure their anonymity.

At the PRM appointments (6 and 12 months after discharge), a detailed physical examination was performed, and a predefined set of scales were applied, aiming to comprehensively evaluate the three domains of PICS (physical, mental, and cognitive). Additionally, patients’ functionality was systematically assessed. The chosen outcome measures were preferentially the ones recommended by the core outcome set for survivors of acute respiratory failure [15].

Physical examination encompassed the evaluation of the patient’s swallowing function, muscle strength, sensory response, signs of CSTD, balance and gait. Data regarding muscle strength, sensory response and signs of CSTD was obtained from physical examination and afterwards formally analyzed through statistical methods. Data regarding swallowing function, balance and gait were analyzed using specific instruments, as described below. Muscle strength was evaluated through manual muscle testing and graded in accordance with the Medical Research Council Sum Score (MRC-SS). The maximum score at this metric is 60 points reflecting maximal strength in all evaluated segments bilaterally, so this value was used as reference category [16]. The sensory exam included the evaluation of light touch sensitivity in all dermatomes from C2-S2 bilaterally. The presence of hyper, hypo or anesthesia, and/or the complains of dys or paresthesia during the sensory examination, were considered as sensory impairments. Signs of CSTD were evaluated through the examination of deep tendon reflexes (DTR) and Babinski sign. Bicipital, tricipital, brachioradialis, patellar and achilles DTR were appraised using a predefined T-shaped reflex hammer. The grading of reflex response was in accordance with an adapted form of the National Institute of Neurological Disorders and Stroke (NINDS) Myotatic Reflex Scale in: 0) absent, 1) hyporeflexia, 2) normal, 3) hyperreflexia, 4) hyperreflexia with unsustain clonus (≤ five beats), 5) hyperreflexia with sustain clonus (> five beats) [17]. Babinski sign was evaluated using the reflex hammer dull point by running up, with light pressure, the lateral plantar side of the foot, from heel to toes. The response of hallux and toes was recorded as extensor (Babinski sign), flexor or neutral [18]. The presence of signs of CSTD was defined as 1) Babinski sign in at least one extremity or 2) hyperreflexia in at least two extremities [19]. All data recorded from the physical examination was included in the physical domain.

To further characterize the physical domain, the European-Quality-of-Life-5-Dimensions-3-Level (EQ-5D-3L) questionnaire was used to measure health-related QoL. The first part of this questionnaire is a three-question component that explores five dimensions: mobility, self-care, usual activities, pain/discomfort and anxiety/depression. Each dimension is rated on a scale from 1 to 3. In accordance with Larsson IM *et al*. study we have considered the result of at least two points per question as cut-off value for impairment (total score ≥ 6) [20]. The second section is a visual analogue scale (EQ-VAS), which is a measure of self-rated overall health status, ranging from 0 to 100%. According to Ferreira PL *et al*. study in the Portuguese population, we have considered values below 75% as an impairment [21].

Fatigue was evaluated using the Portuguese version of Fatigue Assessment Scale (P-FAS) [22]. The P-FAS is a self-reported, 10-item ordinal questionnaire that varies from 10 to 50. A total P-FAS score ≥ 22 indicates the presence of fatigue [23].

Swallowing function was evaluated using the Functional Oral Intake Scale (FOIS) and the Portuguese Eating Assessment Tool (P-EAT-10) [24, 25]. FOIS is a continuous scale that ranges from 1 to 7. In accordance with Sassi FC *et al*. analysis, we divided patients in 1) resolved dysphagia, when FOIS levels were of 6 or 7; or 2) non-resolved dysphagia, if the FOIS levels were from 1 to 5 [26]. The P-EAT-10 is a continuous scale, that varies from 0 to 40, with scores over 3 in each question indicating increase risk of dysphagia, as detailed in Zhang PP *et al*. metanalysis [27]. In accordance, we have considered 30 as the reference value for dysphagia and 0 as the reference for the absence of swallowing complains.

Gait was analyzed and described during physical examination and classified in accordance with the Hauser Ambulation Index (HAI) for analytic purposes [28]. This index ranges from 0 to 9. We have considered 0 as the reference category, thereby classifying patients with scores ≥ 1 as having a gait impairment.

Balance was evaluated objectively in the physical examination and classified accordingly to the Berg Balance Scale (BBS). The BBS assesses the functional balance based on 14 items, with a maximum score of 56 points. In accordance, we have considered that scoring ≤ 55 points was suggestive of having a balance impairment [29]. Additionally, we have considered the cut-off for higher risk of falls (46 points) as reference for the presence of a significative balance impairment [30].

To summarize the obtained results, we have created a composite variable (PICS-physical) that included all subdomains related to the physical function. We have considered that there was an involvement of the physical domain when any of the subdomains was altered.

For the mental domain, we used the Hospital Anxiety and Depression Scale (HADS). We considered 0 points as reference value, thought significant anxiety was defined as HADS-anxiety score ≥ 8 and significant depression as HADS-depression score ≥ 8 [31]. When significant depression or anxiety was reported, an impairment on the mental domain as assumed.

To evaluate the cognitive domain, the Montreal Cognitive Assessment (MoCA) was applied. This score range is 0–30. A cut-off of 26 was used to define cognitive dysfunction in accordance with the literature [32].

Functionality was evaluated through self-reported Functional Independence Measure (FIM). The FIM is a validated and objective assessment of functional status [33]. It is an 18-item ordinal scale, in which the global score varies from 18 to 126 [33]. Since multidisciplinary FIM assessment was not possible in this setting, we have considered self-reported FIM values following previous reports of moderate agreement between self-reported and observed FIM [34]. When the total punctuation at FIM was below 126 points, we considered that the patient was not fully independent on ADL. Even though PICS original definition allocated autonomy on ADL in the physical domain, in our analysis functionality was evaluated separately, since mental, physical and cognitive dysfunction can impact functionality and autonomy on ADL.

Furthermore, information regarding PRM interventions was also collected. Through the ECR, data regarding in-hospital (ICU and wards) rehabilitation programs was retrieved. In each follow-up visit, information concerning PRM programs performed after hospital discharge was obtained, namely, setting (in or outpatient), modalities (uni or multimodal, including rehabilitation nursing, physical therapy, occupational therapy, speech therapy and/or neuropsychology), length (number of months under PRM intervention) and intensity (number of sessions per week and each session duration).

### Statistical analysis

Statistical analysis was performed using Statistical Package for the Social Sciences (SPSS) statistics software (version 27).

Categorical variables are summarized as frequencies and percentages. Continuous variables are summarized as means and standard deviations (variables with normal distribution) or medians and interquartile ranges (variables with skewed distributions). Normal distribution was checked using histogram visual inspection and the Shapiro–Wilk test.

The chi-square test or Fisher’s exact test was used, as appropriate, to compare categorical variables. Continuous variables comparison between “included” and “lost to follow-up” patients was performed with independent sample t-test or the Mann-Whitney U test, according to the variable distribution.

To compare patient’s values with normative data (reference category), one sample t-test or one sample Wilcoxon Signed Rank Test were used, in accordance with the variable distribution. Due to the absence of specific cut-offs for the applied scales in this post-critical COVID-19 sample, and the diversity of results (regarding the used metric and the timing of examination) available in the literature, we compared our results with the classic normative values of each scale, as previously referred as the reference category.

Continuous variables were compared regarding its evolution throughout the 6 month follow-up period using paired-samples t-test or related samples Wilcoxon Signed Rank Test, in accordance with the variable distribution.

All reported p values are two-tailed, with a p-value <0.05 indicating statistical significance.

## Results

### Sample characteristics

A total of 92 critical COVID-19 patients were admitted and discharged alive from our ICU, between May/2020 and September/2021.

Within this eligible sample, 5 patients died after ICU discharge (5.4%), 35 refused to be observed in the appointment (38%) and 10 patients were not reachable (10.9%). As so, 42 patients were included (45.7%). No differences were found between patients included and those lost to follow-up regarding socio-demographic characteristics (gender and age), the severity of disease at admission (APACHE and SAPS II scores), number of days at the ICU and total length of stay (S1 Table).

In the included sample, 66.7% were males with a mean age of 62 years old (standard deviation (SD) = 13.5). All patients were previously independent on ADL. Obesity (57.1%), hypertension (54.8%), and hyperlipidemia (45.2%) were the comorbidities more frequently identified.

The mean SAPS II score was 40.7 (SD = 15.3), with 73.8% (n = 31) of patients scoring over 30 points at ICU admission. Moreover, the median of ICU stay was 31.5 days (interquartile range (IQR) = 15.5–51.3) and the median number of days under IMV was 25 (IQR = 10.0–43.0). During ICU stay, several complications were registered, namely neurological (54.8%), cardiovascular (14.3%), abdominal (42.9%), cutaneous (47.6%) and infectious (71.4%).

Data regarding socio-demographic characteristics, comorbidities, characterization of critical respiratory illness and complications at ICU are detailed in Table 1.

**Table 1:** Socio-demographic and clinical characteristics of the sample.

Characteristic	Total cohort (n = 42)
Socio-demographic characteristics	
Male gender, n(%)	28 (66.7)
Age, mean (SD)	61.8 (13.5)
Previous autonomy on ADL, n (%)	42 (100.0)
Comorbidities	
Hypertension, n(%)	23 (54.8)
Diabetes *Mellitus*, n(%)	18 (42.9)
Hyperlipidemia, n(%)	19 (45.2)
Obesity, n(%)	24 (57.1)
Smoking habits, n(%)	1 (2.4)
Atrial fibrillation, n(%)	3 (7.1)
Ischemic heart disease, n(%)	1 (2.4)
Heart failure, n(%)	1 (2.4)
Peripheral vascular disease, n (%)	3 (7.1)
CPOD, n(%)	1 (2.4)
Asthma, n(%)	5 (11.9)
Sleep apnea, n(%)	6 (14.3)
Psychiatric pathology, n(%)	7 (16.7)
Oncologic pathology, n(%)	7 (16.7)
Immunosuppression, n(%)	1 (2.4)
Characteristics regarding the critical respiratory illness	
APACHE II, mean (SD)	18.1 (5.7)
SAPS II, mean (SD)	40.7 (15.3)
SAPS II ≥ 30, n (%)	31 (74.0)
Days at ICU, median (IQR)	31.5 (15.5–51.3)
Total length of stay, median (IQR)	41 (28.0–81.5)
Number of days under IMV, median (IQR)	25 (10.0–43.0)
Need for vasopressor support, n (%)	30 (71.4)
Number of days under vasopressors, median (IQR)	7 (2.0–21.5)
Need for renal replacement therapy, n (%)	7 (16.7)
Number of days under renal replacement therapy, mean (SD)	27 (14.9)
Need of ECMO, n (%)	5 (11.9)
Number of days under ECMO, mean (SD)	27.8 (30.4)
Need of prone positioning, n (%)	27 (64.3)
Complications during ICU stay	
Neurological complications^¦^, n (%)	
Aphasia, dysarthria or dysphonia	0 (0.0)
Dysphagia	5 (11.9)
Focal weakness	0 (0.0)
Delirium	13 (31.0)
Seizures	0 (0)
Cerebrovascular disease	1 (2.4)
Encephalopathy	2 (4.8)
Encephalitis or myelitis	0 (0.0)
Peripheral neuropathy	2 (4.8)
Signs of CSTD	11 (26.2)
Composite of neurological signs, symptoms, or syndromes	23 (54.8)
Cardiovascular complications^¥^, n (%)	6 (14.3)
Abdominal complications^§^, n (%)	18 (42.9)
Cutaneous complications (pressure injuries), n (%)	20 (47.6)
Infectious complications, n (%)	30 (71.4)

APACHE: Acute Physiology and Chronic Health Evaluation; ECMO: extracorporeal membrane oxygenation; ICU: Intensive Care Unit; IQR: Interquartile range; CPOD: Chronic Pulmonary Obstructive Disease; CSTD: Corticospinal tract dysfunction; SAPS: Simplified Acute Physiology Score; SD: Standard deviation.

^¦^: Neurological complications included aphasia, dysarthria, dysphonia, dysphagia, focal weakness, delirium, seizures, cerebrovascular disease, encephalopathy, encephalitis, myelitis, peripheral neuropathies and signs of CSTD. The composite of neurological signs, symptoms, or syndromes considered, for each patient, the presence of at least one neurological sign, symptom, or syndrome, regardless of the number of neurological manifestations.

^¥^: Cardiovascular complications included bradyarrhythmia, tachyarrhythmia (atrial fibrillation, flutter, other tachyarrhythmias), tachycardia-bradycardia syndrome, secondary myocardial injury, cardiac arrest, pericarditis, pericardial effusion, endocarditis, acute heart failure, and cardiogenic chock.

^§^: Abdominal complications included hepatitis, elevated liver enzymes, gastrointestinal bleeding, pseudo-obstruction and obstruction, diarrhea, and constipation.

### PRM care characterization

Throughout ICU and hospital wards stay, all included patients were evaluated and included in PRM inpatient programs, which consist of one (physical therapy, occupational therapy, speech therapy, and rehabilitation nursing) or more treatment modalities, pending on each patient sequelae. After discharge, 88% (n = 37) maintained PRM treatments: 7 patients (16.7%) were admitted at PRM facilities for inpatient rehabilitation (followed by outpatient rehabilitation), and the remaining 30 patients were included in outpatient rehabilitation programs. A total of 5 patients were not included in PRM programs after hospital discharge since a full recovery in neuromotor domains and autonomy on ADL was achieved. In most cases, PRM outpatient intervention was unimodal (physical therapy; 70.2%; n = 26). The frequency of PRM interventions ranged between 2 to 5 sessions per week, in which each session length of 30 to 60 minutes. At 6 months post-discharge, 62.2% remained under PRM intervention, while at 12 months 32.4% maintained PRM outpatient programs.

### PICS analysis

Table 2 details the clinical status at 6 and 12 months, univariate analysis between the achieved scores and the reference values and a comparative analysis (“evolution” between scores at 6 and 12 months.

**Table 2:** Scores at 6 and 12 months and comparison with normative values.

	6 months			12 months		p-value (evolution)
	Status		p-value (comparison with reference)	Status	p-value (comparison with reference)	
Physical Domain						
Quality of Life						
EQ-5D-3L, median (IQR) *Reference*: 6 points	7.2 (5.0–8.5)	0.034^*a*^		6.4 (5.0–7.0)	0.180^*a*^	0.069^*c*^
EQ-VAS, mean (SD) *Reference*: *75%*	67.4 (21.6)	<0.060^*a*^		73.3 (18.5)	0.573^*a*^	0.038^*c*^
Fatigue						
FAS: mean (SD) *Reference*: *22 points*	34.5 (32.0)	0.009^*b*^		18.5 (50.0)	0.309^*b*^	0.043^*d*^
Swallowing function						
FOIS: median (IQR) *Reference*: *5 points*	7.0 (7.0)	0.157^*b*^		7.0 (7.0)	1.000^*b*^	0.157^*d*^
EAT10: median (IQR) *Reference*: *0 points*	0.0 (0.0)	0.180^*b*^		0.0 (0.0)	0.317^*b*^	0.285^*d*^
Muscle strength						
MRC-SS: median (IQR) *Reference*: *60 points*	60.0 (48.0–60.0)	0.001^*b*^		60 (56.8–60.0)	0.002^*b*^	0.003^*d*^
Gait ability						
HAI: median (IQR) *Reference*: *0 points*	1.0 (0.0–2.3)	<0.001^*b*^		0.0 (0.0–1.0)	<0.001^*b*^	0.026^*d*^
Balance						
BBS: median (IQR) *Reference*: *56 points*	51.0 (42.3–56.0)	<0.001^*b*^		56 (50.0–56.0)	<0.001^*b*^	0.010^*d*^
Sensibility						
Impairment: n (%)	7.0 (16.7)			3.0 (7.1)		0.331^*e*^
Signs of CSTD						
Present: n (%)	22.0 (52.3)			11.0 (26.2)		<0.001^f^
Phycological Domain						
Anxiety and depression						
HADS–Anxiety: mean (SD) *Reference*: *0 points*	5.2 (4.5)	<0.001^*a*^		4.0 (4.3)	<0.001^*a*^	0.012^*c*^
HADS–Depression: mean (SD) *Reference*: *0 points*	6.3 (3.7)	<0.001^*a*^		5.2 (4.9)	<0.001^*a*^	<0.001^*c*^
Cognitive Domain						
Cognitive Performance						
MoCA: median (IQR) *Reference*: *26 points*	20.0 (11.3–24.8)	<0.001^*b*^		23.0 (16.0–26.5)	<0.001^*b*^	0.031^*d*^
Functionality and autonomy in daily-life activities						
FIM: median (IQR) *Reference*: *126 points*	126.0 (125.0–126.0)	0.018^*b*^		126.0 (126.0)	0.018^*b*^	0.345^*d*^

BBS: Berg Balance Scale; CSTD: Corticospinal tract dysfunction; EAT-10: Eating Assessment Tool; FAS: Fatigue Assessment Scale; FIM: Functional Independence Measure; FOIS: Functional Oral Intake Scale; HADS: Hospital Anxiety and Depression Scale; HAI: Hauser Ambulation Index; IQR: Interquartile range; MoCA: Montreal Cognitive Assessment; MRC-SS: Medical Research Council Sum Score; SD: Standard deviation

^*a*^ *One sample t-test;*

^*b*^ *One sample Wilcoxon Signed Rank Test;*

^*d*^ *Paired-samples t-test;*

^*e*^ *Related Samples Wilcoxon Signed Rank Test;*

^*f*^ *Fisher Exact Test;*

^*g*^ *Chi-square test*.

### PICS—Physical domain

PICS physical domain was evaluated through multiple metrics in our analysis. Indeed, 76.2% (n = 32) of our sample had at least one physical impairment at 6 months decreasing to 71.4% (n = 30) at 12 months (p-value = 0.475).

Regarding QoL, at 6 months after ICU discharge, COVID-19 survivors had significantly higher EQ-5D-3L median (7.2 (IQR = 5.0–8.5), p-value = 0.034), and a lower mean EQ-VAS (67.4 ± 21.6, p-value = 0.060), in comparison with reference values. Nevertheless, one year after ICU discharge, we found no difference either in EQ-5D-3L nor in EQ-VAS compared to normative values (p-value = 0.180 and p-value = 0.573, respectively). In both QoL metrics, there was a tendency for improvement through the follow-up period, which was statistically significant only for the EQ-VAS (p-value = 0.038).

Concerning fatigue, COVID-19 survivors had a mean P-FAS of 34.5 ± 32.0 at 6 months, a significantly superior value in comparison with reference values (p-value = 0.009). At 12 months, the median FAS decreased to 18.5 points (SD = 50.0; p-value = 0.043).

Swallowing function was also analyzed in our sample, as part of the physical domain. Both at 6 and 12 months, the median of FOIS and P-EAT-10 were similar to the reference category. At 6 months after ICU discharge, 5% (n = 2) presented swallowing impairments whereas at 12 months none had this complication.

A total of 31% of COVID-19 survivors had muscle weakness at 6 months, and 28.6% maintained this impairment at 12 months (p-value = 0.002). In brief, both at 6 and 12 months after ICU discharge, median values at MRC-SS were significantly below the normative value (6 months: median = 60.0, IQR = 48.0–60.0, p-value = 0.002; 12 months: median = 60.0, IQR = 56.8–60.0, p-value = 0.001). Nonetheless, during the follow-up period, there was a significant improvement on this parameter (p-value = 0.003).

Gait, assessed objectively through the HAI, was also significantly impaired, in comparison with reference values, both at 6 and 12 months after ICU discharge (6 months: median = 1.0, IQR = 0.0–2.3, p-value<0.001; 12 months: median = 0.0, IQR = 0.0–1.0, p-value<0.001). Similarly, there was a significative improvement in this subdomain through the follow-up period (p-value = 0.026).

Regarding functional balance, median BBS was 51.0 (IQR = 42.3–56.0) at 6 months and 56.0 (IQR = 50.0–56.0) at 12 months, values that were significantly reduced in comparison with the reference category (p-values<0.001). Nevertheless, this parameter also evolved positively through time (p-value = 0.001). Moreover, when analyzing the BBS through a cut-off for higher risk of falls, at 6 months 28.6% had a higher risk of this adverse event, a value that decreased to 18.9% at 12 months (p-value = 0.004).

Sensory impairments were present in 16.7% at 6 months and in 7.1% at 12-months. No significant improvement on this subdomain was depicted through the study period (p-value = 0.331).

Signs of CSTD were present in 52.3% (n = 22) at 6 months, and in 26.2% (n = 11) at one year follow-up (p<0.001).

### PICS–Mental domain

Overall, at 6 months after ICU discharge, 31.0% (n = 13) had impairments on the mental domain of PICS, value that was reduced to 26.2% (n = 11) at 12 months (p-value = 0.159).

Both at 6 and 12 months after ICU discharge, the mean score at the HADS–Anxiety (5.2±4.5 and 4.0±4.3, respectively) and at the HADS–Depression scores (6.3±3.7 and 5.2±4.9, respectively) was significantly superior to the reference value (p-value<0.001). Using the 8 points cut-offs for significant anxiety and depression, a significant difference from our values was also reported (p-value = 0.003 and 0.022), pointing to the absence of significant impairments, despite the presence of alterations. In both anxiety and depression scores, there was a significative positive evolution through the follow-up period (anxiety: p-value = 0.012; depression: p-value<0.001).

### PICS—Cognitive domain

The prevalence of cognitive dysfunction at 6 months after ICU discharge was of 79.2%, value that shifted to 64.9% at 12-months (p-value = 0.003). In fact, significant disablement was noted in comparison with reference values both at 6 months (median = 20.0, IQR = 11.3–24.8, p-value<0.001) and 12 months (median = 2.03; IQR = 16.0–26.5; p-value<0.001). Yet, a global improvement in this 1-year follow-up was noted (p-value = 0.0031).

### Functionality

In our sample, 54.8% of the patients were fully active on ADL 6 months after ICU discharge, with a median FIM of 126.0 (IQR = 125.0–126.0), a value significantly inferior to the reference category (p-value = 0.018). Moreover, at 12-months after ICU discharge, functionality significantly improved, with 74% of patients being totally independent (p-value = 0.002). Nevertheless, median FIM was still significantly diminished (median = 126.0, IQR = 126.0, p-value = 0.002).

## Discussion

Our study revealed that critical COVID-19 survivors present substantial physical, mental and cognitive impairments 6 and 12 months after ICU discharge, and that these impairments seem to improve through time. Nevertheless, 1 year after ICU discharge, significant disablements in muscle strength, gait ability, balance, psycho-emotional status and cognitive performance persisted. Approximately half of these survivors were fully independent on ADL 6 months after ICU discharge, value that improved by approximately 20% on the subsequent 6 months.

Previous studies have described persistent signs, symptoms and reduced health related QoL after COVID-19 disease in hospitalized patients [10–13]. Irisson-Mora I *et al*. suggested that the grade of severity of disease (critically ill *vs*. hospitalized patients) impacts the prevalence of impairments in COVID-19 survivors [35]. Nonetheless, studies investigating specifically critically ill COVID-19 survivors are still scarce and lack on multidomain assessments and clinical path analysis. Previous investigations were generally limited to describing outcomes in specific time frames, without exploring clinical trajectories [36, 37] or assessing global patient status, not having in consideration PICS main domains and its’ impact on functionality [10, 11]. Additionally, part of these studies’ methodology included telephone interviews, leading to an absence of physical examination data and to additional biases [10, 11, 37]. Furthermore, most studies that included face-to-face interviews focused on the respiratory and cardiovascular sequelae of critical COVID-19 disease, and not in the physical and cognitive impairments [13, 38]. Our investigation is, to our knowledge, the first to provide a comprehensive analysis of PICS domains trajectory in critical COVID-19 survivors, including not only scales but also physical examination and functionality data.

PICS is known to be a common syndrome after critical care. In this COVID-19 era, due to the marked increase in ICU admissions, the number of patients suffering from this syndrome is rising. Also, critical COVID-19 patients may be particularly prone to develop PICS [39]. Firstly, PICS risk factors are frequent among the COVID-19 critical patient [40]. Secondly, since median ICU and hospital length of stay are usually longer in this population, the subsequent prolonged bed rest and extended hospital stay may contribute to muscular weakness, which is associated with substantial impairments in physical function and health related QoL [41]. Despite the possible increased risk for PICS in the survivors of critical COVID-19, the prevalence of PICS and its definition for this population is still not yet determined [35]. Hence, the possible differential extent and impact of PICS in COVID-19 survivors, in comparison with non-COVID-19 ICU survivors, warrants further clarification. Hodgson CL *et al*. performed a comparative analysis between critical COVID-19 and non–COVID-19 survivors, reporting that the incidence and severity of disabilities, health related QoL, psychological status and cognitive performance at 6 months did not significantly differed between COVID-19 and non–COVID-19 survivors [37]. Nevertheless, this study included a non-matched sample of patients from two different prospective cohorts, in which there were some baseline differences that could affect the results. Also, this study included a single telephone evaluation, so data regarding physical examination and information regarding the clinical path was not included. On the other hand, in-ICU studies, as Rahiminezhad E *et al*., that compared critical COVID-19 and non-COVID-19 patients regarding functional parameters, reported significantly higher disability in COVID-19 patients [42]. Also, the RECOVID study pointed to a quicker recovery in COVID-19 patients in comparison with patients with other ICU admission motives [43]. Therefore, the real impact of critical COVID-19 on the prevalence, extent and characteristics of PICS warrants further clarification, as this population may have specific needs and different clinical courses.

The baseline characteristics of our sample, namely socio-demographic factors, comorbidities and the characteristics related to the critical respiratory illness, were compatible with previous investigations in this field [10, 37]). We highlight that the mean age of our sample was of 61.8 years old, ranging from 28 to 81 years old. This data is in line with most previous investigations in this field [4, 6, 11–13, 44]. Nevertheless, and as advanced age is not only a risk factor for higher severity and mortality due to COVID-19, but also a predictor of PICS, we stress that our study data and conclusions should be considered as driven for a cohort of older adults [45, 46].

Regarding PRM intervention, all included patients were evaluated and included in intra-hospitaller PRM programs and all patients who had clinical indication maintained these interventions after hospital discharge. Our data is in line with previous studies, specifically concerning the setting and median duration of PRM programs [35]. The transversal inclusion on PRM programs in this sample, and the maintenance of these interventions for long periods, may be part of the explanation for the significative PICS improvement. Indeed, Berentschot JC *et al*. multicenter prospective cohort study highlighted the impact of post-discharge PRM programs in several physical domains, specifically when performed in multi-modal and comprehensive settings [47]. Nevertheless, direct comparative analysis between different PRM programs regarding its setting, modalities, duration, intensity and other characteristics, still lacks in the hitherto literature. As we have performed an observational analysis, and not an experimental or quasi-experimental study, a clear analysis of the impact of PRM intervention in the multidomain improvement on this sample was not possible to perform. Indeed, it would be clinically relevant to access the specific contribution of PRM intervention in these patients’ clinical path, differentiating the rate of improvement that is indexed to the diseases’ natural course from the induced by PRM intervention. Nonetheless, our findings emphasize the long-term impact of critical COVID-19, with clear implications for clinical care, specifically in the field of PRM.

In our sample, COVID-19 survivors had significative impairments on QoL at 6 months, with a significative recovery during the first year after ICU discharge. In comparison with Huang L *et al*. study, EQ-VAS was similar at 1-year, yet at 6 months lower scores were noted in our sample [11]. Similarly, significative fatigue at 6 months was noted, which positively evolved until the first year after ICU discharge. In comparison with Hussain N *et al* analysis, our data points out to slightly lower prevalence of this symptom [48].

Swallowing function was also analyzed, as part of the physical domain. Nonetheless, it was not possible to compare our data with follow-up analysis of critical COVID-19 survivors at 6 and 12 months in relation with its absence in the literature.

Muscle strength, gait and balance were also significantly impaired in our sample, both at 6 and 12 months, despite the significantly positive evolution reported through the follow-up period. Since previous studies did not measure these domains, neither at the same timings nor with the same instruments, a comparative analysis with other ICU (ideally COVID-19) studies was not possible.

Sensory impairments were not common in this sample and did not improve significantly over time. Due to the more subjective nature of sensory complains, clinical assessment may have underestimated the prevalence of these complication or its clinical improvement. Most previous studies that have addressed this impairment included only subjective complains and not objective analysis [49]. Nevertheless, according to Pinzon RT *et al*. meta-analysis, sensory impairments were reported in one in each three COVID-19 survivors, a value that exceeds our data by around 50% [49]. The inclusion of specific sensory examinations in follow-up analysis of critical COVID-19 survivors lacks in the literature, which seems to be a significative flaw as a result of the negative impact of these alterations [50].

Regarding signs of CSTD, none of the previous analysis of mid- and long-term morbidities after critical illnesses have analyzed this manifestation. As so, a direct comparison with a literature was not viable. Nevertheless, there are several reasons to justify the formal search for signs of CSTD. Firstly, these analysis allow a rapid distinction between upper and lower motor neuron pathology [51]. Secondly, as magnetic resonance imaging studies of COVID-19 patients showed that corticospinal tract lesions were the most common lesions of the white matter, CSTD evaluation may raise clinical suspicion of neurological impairments, allowing earlier diagnosis and treatment [52]. Lastly, as clonus can have a direct impact on gait ability, and therefore influence the patient’s functionality, its’ active exploration is warranted in the setting of PRM outpatient appointments and therefore should be considered in subsequent investigations that aim to access neurological and functional consequences of critical COVID-19. We have compared this sample rates with the prevalence of CSTD signs in our group cohort of critical COVID-19 patients [53]. We highlight that, despite similar clinical methodology, material and diagnostic criteria, higher rates of CSTD were encountered at 6-months after ICU discharge, which is probably in relation with the impact of intensive care unit acquired weakness on DTR response (its diminishment or abolition) [54]. Nevertheless, the number of patients with signs of CSTD significantly decreased through the follow-up period, highlighting the probably neurological recovery throughout the first year after ICU discharge.

PICS mental domain involvement was inferior when compared to the physical domain in our analysis. Yet, one in each four patients at 1-year maintained psychological complains, data compatible with the literature [9].

Poor cognitive performance after COVID-19 has been previously reported [37]. The proportion of patients who recovered cognitive function over time is in line with previous reports [10]. Nonetheless, in our sample, significative cognitive impairments persisted at 12 months after ICU surpassing previous data from Taniguchi *et al*. study [10]. Miskowiak KW *et al*. postulated that cognitive impairments were associated with the degree of long-term pulmonary dysfunction, increased respiratory symptoms and D-dimer concentrations during acute illness, suggesting a potential link to restricted oxygen delivery to the brain [40]. Since our sample comprises a severe cohort of COVID-19 critically ill patients, the overexpression of this cognitive impairments is most likely in this context.

Overall, PICS was prevalent in our study population, both at 6 and 12 months after ICU discharge. Since there is a significative heterogeneity on PICS diagnostic criteria, direct comparisons between our data and the literature are hard to establish [9]. When analyzing the prevalence of each component of PICS, the main contribution for mid- and long-term impairments was the physical domain, which was confirmed in up to three-fourths of the sample both at 6 and 12 months, data compatible with the literature [12]. Moreover, we highlight the continued recovery of PICS domains throughout our follow up period, data already hypothesized by Zhang H *et al*. [55].

The study design and methodological strengths reinforce our major findings. A prospective cohort study was designed as it clearly indicates the temporal sequence between exposure and outcome, and allows a better characterization of the clinical path. Moreover, this study design allows the examination of multiple effects of a single exposure (namely, and applying to this investigation, the effects of critical COVID-19 on physical, cognitive and mental domains) [56]. To ensure the external validity of our results, there was a consecutive sampling of participants and inclusion of patients from different ICU. To increase patients’ engagement, thereby reducing the rates of “lost to follow up”, several telephone trials were attempted to recruit the patients, and each patient that missed an appointment was given up to two opportunities to reschedule the visit. Indeed, and in accordance with a prospective cohort study design, the risk of “losses to follow up” was not negligible. We also highlight that a differential loss to follow up could have introduced additional biases. Hence, the rates of “lost to follow up” of our study were in line with previous studies [55]. Moreover, “included” and “lost to follow-up” patients were compared through statistical analysis, emphasizing the external validity of our data. Regarding the internal validity, we stress that all patients were evaluated by a single investigator (a PRM physician), using validated instruments, and thereby ensuring high-quality data and minimizing bias.

Our study presents some limitations. Firstly, we developed a single-center cohort study. The local case-mix may have influenced our results, and its generalization might be limited. Nevertheless, as the study took place in a tertiary care center covering a wide geographical referral area, encompassing several specialized ICU, and receiving several patients transferred from other institutions for advanced ICU care, the external validity of our results is, hence, considerable. Furthermore, and in line with a cohort study design, variables and outcomes were only observed, without any intervention taking place [56]. Secondly, we did not have a contemporaneous control group of critically ill respiratory patients without COVID-19 infection, so we could not distinguish the specific long-term effects of this infection from those that might result from critical illness itself. Also, sample size calculation was not performed due to the lack of data on the chosen outcome measurements available at the literature by the time our recruitment started. Lastly, all patients were evaluated by PRM physicians during hospitalization, and most were included in tailored PRM treatments, which lead to an inability to estimate the impact of PRM programs on PICS trajectory.

Further studies are warranted to characterize the long-term trajectory of PICS in critical COVID-19 patients. Also, predictive models for PICS diagnosis and prognosis are desirable. Moreover, the promising effects of vaccines and other new treatments in PICS need further description in COVID-19 survivors. Furthermore, additional studies, preferably of an experimental nature, designed to assess the impact of different settings, modalities and duration of PRM programs are desirable to further determine the most effective PRM programs in this setting. Finally, future studies should explore the link between brain oxygen delivery and cognitive outcomes and therapies that may attenuate the effect of acute respiratory failure on cognitive impairment.

## Conclusion

Critical COVID-19 survivors present significative physical, mental and cognitive impairments 6 and 12 months after ICU discharge, despite their positive evolution through time. Accordingly, at least during the first-year post ICU discharge, but probably for a longer period, COVID-19 patients benefit from PRM evaluations and interventions, since clinical and functional impairments persist.

## Supporting information

S1 Table Comparison between “included” and “lost to follow-up” patients. (DOCX)
