# Supplementary material for: Surviving critical COVID-19: How functionality, physical, mental and cognitive outcomes evolve?
Source: PLoS One. 2023 Jun 23;18(6):e0284597. doi: 10.1371/journal.pone.0284597 (PMC10289386; doi:10.1371/journal.pone.0284597)
Supplement: S1 Table — (DOCX) [file pone.0284597.s001.docx]

**S1 Appendix**

| Table S1. Comparison between “included” and “lost to follow-up” patients. | | | |
| --- | --- | --- | --- |
|  | Included (n=42) | Lost to follow-up (n=49) | p-value |
| Male gender, n (%) | 28 (66.7) | 35 (71.4) | 0.624 ^a^ |
| Age, mean (SD) | 61.8 (13.5) | 61.7 (12.5) | 0.967 ^b^ |
| APACHE, mean (SD) | 18.1 (5.7) | 18.3 (6.0) | 0.857 ^b^ |
| SAPS II, mean (SD) | 40.7 (15.3) | 39.6 (14.5) | 0.726 ^b^ |
| Days at ICU, median (IQR) | 31.5 (15.5-51.3) | 26.5 (17.8-50.0) | 0.978 ^c^ |
| Total length of stay, median (IQR) | 41 (28.0-81.5) | 46 (28.0-65.5) | 0.884 ^c^ |
| Legend: *APACHE: Acute Physiology and Chronic Health Evaluation; ICU: Intensive Care Unit; IQR: Interquartile range; SAPS: Simplified Acute Physiology Score; SD: Standard deviation; ^a^ Chi-square test; ^b^ independent sample t-test; ^c^ Mann-Whitney U test* | | | |
